# Dengue encephalopathy in an adult due to dengue virus type 1 infection

**DOI:** 10.1186/s12879-024-09198-z

**Published:** 2024-03-15

**Authors:** Xingyu Leng, Huiqin Yang, Lingzhai Zhao, Jiamin Feng, Kanghong Jin, Lu Liao, Fuchun Zhang

**Affiliations:** 1 Guangzhou Medical Research Institute of Infectious Diseases, Department of Infectious Disease, Guangzhou Eighth People’s Hospital, Guangzhou Medical University, 510000 Guangzhou, P.R. China; 2 Guangzhou Medical Research Institute of Infectious Diseases, Department of Clinical Laboratory, Guangzhou Eighth People’s Hospital, Guangzhou Medical University, 510000 Guangzhou, P. R. China; 3 Guangzhou Medical Research Institute of Infectious Diseases, Institution of Infectious Disease, Guangzhou Eighth People’s Hospital, Guangzhou Medical University, 510000 Guangzhou, P.R. China

**Keywords:** Dengue, Encephalopathy, DENV serotype 1, Adult, Cytokines

## Abstract

**Background:**

Dengue is an important public health problem, which caused by the dengue virus (DENV), a single-stranded RNA virus consisted of four serotypes. Central nervus system (CNS) impairment in dengue usually results from DENV-2 or DENV-3 infection, which lead to life-threatening outcomes. Furthermore, neurological complications due to DENV-1 was rare especially in adult patients.

**Case presentation:**

A 44-year-old man without comorbidities had lethargy after hyperpyrexia and a positive DENV NS1 antigen was detected for confirming the diagnosis of dengue on day 8 of onset. Then logagnosia, decreased muscle strength, delirium and irritability were occurred even radiographic examination were normal. He was treated with low-dose hormone, sedatives and gamma goblin with a short duration of 6 days. The cerebrospinal fluid (CSF) tests were persistent normal. However, presence of DENV-1 RNA was confirmed both in CSF and serum. Furthermore, the complete sequence of the DENV isolated from the patient’s serum was performed (GenBank No.: MW261838). The cytokines as IL-6, IL-10 and sVCAM-1 were increased in critical phase of disease. Finally, the patient was discharged on day 24 of onset without any neurological sequelae.

**Conclusion:**

Encephalopathy caused by a direct CNS invasion due to DENV-1 during viremia was described in an adult patient. Treatment with low-dose hormone and gamma goblin was helpful for admission.

**Supplementary Information:**

The online version contains supplementary material available at 10.1186/s12879-024-09198-z.

## Background

Dengue is an acute infectious disease caused by the dengue virus (DENV), which has been endemic in more than 100 countries recently [1, 2]. The World Health Organization (WHO) has announced that dengue is one of the ten threats to global health in 2019 [3]. DENV was a *Flavivirus* which was divided into four serotypes named 1 to 4 according to their different antigenicity, and each serotype marked several genotypes according to sequence variation [4, 5]. Dengue has a broad spectrum of clinical symptoms, ranging from mild dengue fever to severe dengue, with the classification of severe hemorrhage, severe plasma leakage, and severe organ involvement. The central nervous system (CNS) involvement was recognized as one of the criteria for severe dengue diagnosis [6]. The incidence of these symptoms ranges from 0.5 to 21% of patients hospitalized with dengue [7–9].

All four serotypes of DENV are known to be neurotropic, but DENV-2 and DENV-3 are reported to be the more common viruses [10–13]. CNS impairment caused by DENV-1 was rare. This report describes a case of an adult with fever, rash and neurological manifestations along with confirmed infection with DENV-1.

## Case presentation

**Fig. 1:**
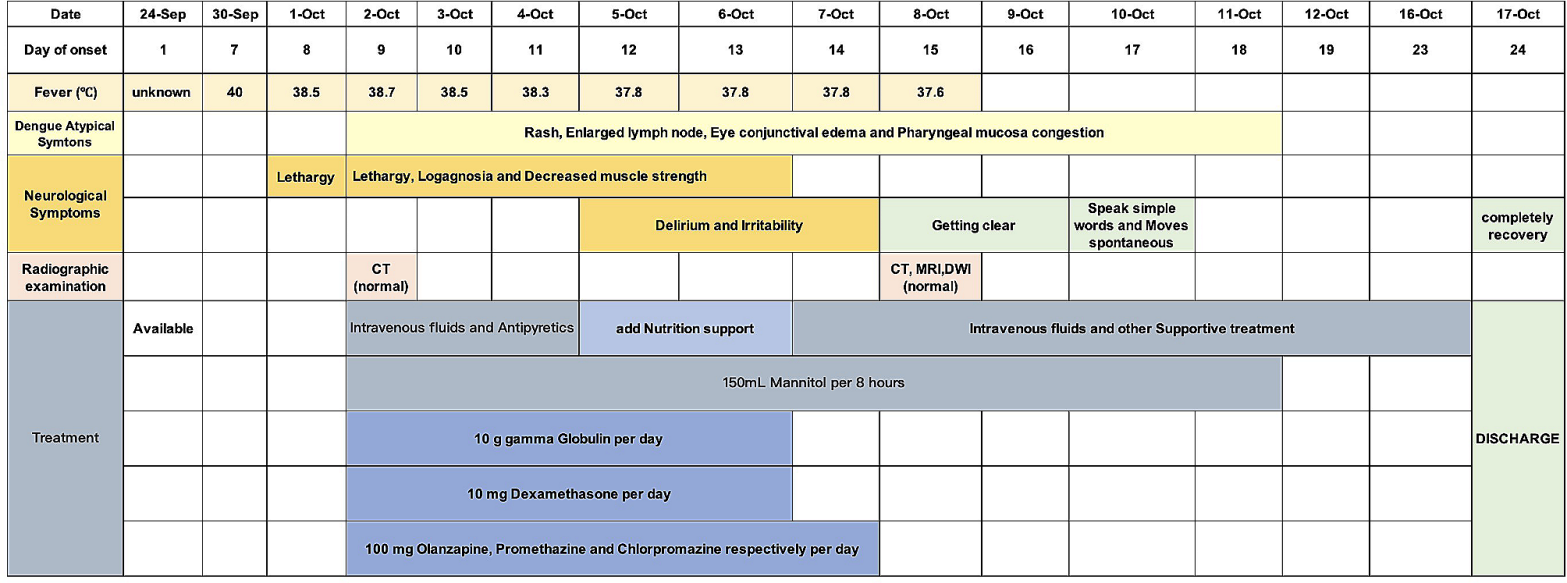
Disease process according to the day of onset between 24 September and 17 October

A 44-year-old man without comorbidities, who lived in Guangzhou, Guangdong province, China, got a fever on 24 September, 2019. After treatment without available details in a clinic, he felt better and got back to work. On 30 September, he had a hyperpyrexia (body temperature was 40℃). One day after, he presented lethargy along with a positive DENV NS1 antigen. Furthermore, he had neurological symptoms like lethargy, logagnosia and decreased muscle strength between 2 and 6 October. The neurological manifestations get worse progressively, delirium and irritability were occurred in 1 and 10 October. However, the radiographic examinations were persistent normal during whole illness (Fig. 1).

Molecular biology test confirmed the presence of DENV in serum during hospitalization and in CSF on day 16 of onset by fluorescent PCR (Daan Gene, Gaungzhou). Both in serum and CSF, DENV-1 RNA was detected precisely (Table 1). Furthermore, DENV was isolated from the patient’s serum. The completed sequence data of isolated DENV strain (GenBank NO.: MW261838) was uploaded to GenBank database after sequencing performed by Sangon Biotech (Shanghai, China). Anti-DENV immunoglobulin G (IgG) and immunoglobulin M (IgM) were negative in serum measured by colloidal gold immunochromatography (Wonfo, Guangzhou) during early hospitalized phase. On the last day of hospitalization, IgM in his serum turned positive. Two followed up visits were made on week 5 and 63 of onset respectively, IgM was persistent negative in the two visits (Table 1).

**Table 1:** Virological features of the case with dengue encephalopathy

Disease phase	Sample	Time of onset	Fluorescent PCR	Virus isolation	DENV serotype	Genome sequencing	DENV genotype	Ig G	Ig M
Hospitalization	CSF	10 d	N	N					
		16 d	P	N	I				
	Serum	11 d	P	N	I				
		15 d	P	P	I	MW261838	I		
		16 d	P	N	I			N	N
		18 d	P	ND	I				
		21 d	P	ND	I				
		24 d	P	ND				N	P
Follow up		5 w						N	N
		63 w						N	N

NOTE: CSF, cerebrospinal fluid; DENV, dengue virus; Ig G, Anti-DENV immunoglobulin G; IgM Anti-DENV immunoglobulin M; N, negative; P, positive; ND, not done.

Cerebrospinal fluid (CSF) analysis on day 10 and 16 of onset were within normal limits, including pressure, color, cell type, cell count, biochemistry and bacteria/fungi culture (sTable 1). Changes in laboratory tests were summarized in sFig. 1. Between the 7 and 9 October, WBC, Lym, HCT and RBC reached the peak while PLT got the minimum value at the same time. The level of serum albumin (ALB) was below 35 U/L since 7 October then rose to the normal range before discharging.

Because lack of effective drugs for dengue, the patient was managed conservatively with dehydration (mannitol, 150mL, per 8 h) intravenous fluids and antipyretics along with close clinical monitoring. During day 9 and 13 of onset, 10 mg dexamethasone and 10 g gamma globulin per day were used in light of infection and weak immunity. From day 9 to 14 of onset, 100 mg olanzapine, promethazine and chlorpromazine respectively per day were helpful for sedative. After the effective treatment, neurological symptoms have alleviated since day 17 of onset. On 17 October, the patient was recovered completely without any neurological sequelae and discharged (Fig. 1).

Additional laboratory examination used to explore the relationship between CNS impairment and immune reaction. The cytokines in serum samples of the patient and a health people (male, 48-year-old) were detected by ELISA. The levels of IL-6, IL-10 and sVCAM-1 were higher than those of health people. Meanwhile, the concentration of cytokines above on day 12 of onset was the highest during the whole illness, and then decreased on day 17 of onset (sTable 2).

## Discussion and conclusion

Although most dengue patients are asymptomatic or mildly symptomatic, about 5% of that can lead to life-threatening severe dengue [14]. According to dengue guidelines published by WHO in 2009, CNS impairment was one of severe organ involvement. Total of four serotype of DENV were confirmed to be neurotropic [10], but DENV-2 and DENV-3 are the most frequently reported as the cause of CNS injury. DENV-1 was associated with neurological complication among pediatric patients in southeast countries [15–17]. Considering DENV-1 was the most predominant serotype of dengue in Guangdong, the largest dengue endemic area in China [18]. Explicit course of neurological complication in adult dengue patients should be helpful for patients admission. The case was diagnosed dengue caused by DENV-1 with definite evidences as (1) DENV NS-1 antigen was detected before hospitalization (2) presence of DENV-1 RNA both in his CSF and serum was confirmed by fluorescent PCR (3) DENV was isolated from his serum (GenBank NO.: MW261838) and (4) positive Ig M was measured in recovery phase.

Dengue associated neurological complications result in prolonged course of disease. Wenxi Hong et al. have reported that the hospitalized duration of adult patients with sever dengue was 10 days [19], however, that of the case we described was 23 days. Definite evidence of DENV-1 RNA measured in the CSF indicated that CNS invasion occurred both in child and adult. Unlike self-limiting neurological disease in pediatric patient infected with DENV-1, the neurological manifestations in adult were getting worse without effective management. Considering lack of effective drugs for dengue, low-dose hormone and sedatives were contributed to alleviate neurological manifestations.

Previous studies reported that the radiographic examination of dengue neurological complications shows spontaneous microhemorrhages, lacunar cerebral infarction and cranial edema due to plasma leakage [20]. However, abnormal findings of radiographic examinations have not seen in the patient. We suggest that patients who present with significant neurological symptoms but no imaging are still administered as patients with CNS involvement.

The mechanism of CNS impairment by DENV was still unknown. Any signs of plasma leakage were not presented in the patient, what accompany with normal HCT level, it suggests that the CNS involvement of the patient was not associated with plasma leakage. Evidence of DENV presented in serum and CSF was confirmed by fluorescent PCR. Furthermore, the time of DENV RNA detection in serum was preceeding. In addition, on the day 10 and 15 of onset, the levels of IL-6, IL-10 and sVCAM-1 in patient’s serum was higher significantly than that in health people. Cytokines both IL-6 and IL-10 could damage the blood brain barrier (BBB) and further facilitate the entry of other immune mediators into the brain subsequently lead to CNS injury [21]. Otherwise, sVCAM-1 is proved to be a marker for endothelial injury under inflammatory processes [22]. These elevated cytokines result in injured BBB, further a direct CNS invasion due to DENV during viremia. Accompany with inflammatory, CNS was damaged. More details in mechanism of CNS impairment by DENV need to explore.

To our best knowledges, it’s a first report about an adult patient with dengue encephalopathy caused by DENV-1, which supported by definite evidences for diagnosis. In conclusion, dengue patient who infected with DENV-1 was also presented neurological complication, but without abnormal radiographic examination. Detection of DENV-1 RNA both in CSF and serum indicates that DENV-1 could invade CNS during viremia. Finally, the patients recovered uneventfully after treatment with low-dose hormone and gamma goblin in time. Elevated cytokines were measured in serum, which may be associated with CNS involvement in adult dengue patient.

### Electronic supplementary material

Below is the link to the electronic supplementary material.

Supplementary Material 1

Supplementary Material 2

Supplementary Material 3

## Data Availability

The dengue virius serotype 1 sequence data that support the findings of this study have been deposited in GenBank with the primary accession code MW261838.
