# Supplementary material for: Dengue encephalopathy in an adult due to dengue virus type 1 infection
Source: BMC Infect Dis. 2024 Mar 15;24:319. doi: 10.1186/s12879-024-09198-z (PMC10943806; doi:10.1186/s12879-024-09198-z)
Supplement: Supplementary file 1 — Supplementary Material 1 [file 12879_2024_9198_MOESM1_ESM.docx]

| **sTable 1 Cerebrospinal fluid test in the case with dengue encephalopathy** | | |
| --- | --- | --- |
| Variables | Day 10 of onset | Day 16 of onset |
| Pressure (mmHg) | 13.5 | 10 |
| WBC (cells/L) | 1.00E+06 | 4.00E+05 |
| RBC (cells/L) | N | N |
| Pandy’s test | N | N |
| Protein (mg/L) | 521.1 | 479.9 |
| Glucose (mmol/L) | 3.14 | 3.58 |
| chlorine (mmol/L) | 125.3 | 115.7 |
| Gram/ Culture | N | N |
| NOTE: WBC, white blood cell; RBC, red blood cell; N, negative. | | |
