# Supplementary material for: Dengue encephalopathy in an adult due to dengue virus type 1 infection
Source: BMC Infect Dis. 2024 Mar 15;24:319. doi: 10.1186/s12879-024-09198-z (PMC10943806; doi:10.1186/s12879-024-09198-z)
Supplement: Supplementary file 2 — Supplementary Material 2 [file 12879_2024_9198_MOESM2_ESM.docx]

| **sTable 2 Cytokines in the case with dengue encephalopathy** | | | | |
| --- | --- | --- | --- | --- |
|  | Day of onset | IL-6 (pg/mL) | IL-10  (pg/mL) | sVCAM-1 (pg/mL) |
| Normal Control | _ | 0.01 | 7 | 10132 |
| Patient | 8 | 19.57 | 30 | 59850 |
|  | 12 | 27.82 | 101 | 146529 |
|  | 17 | 24.53 | 47 | 106214 |
| NOTE: IL, Interleukin; sVCAM-1, soluble vascular cell adhesion molecule 1. | | | | |
