# Supplementary figures and images for: Dengue encephalopathy in an adult due to dengue virus type 1 infection
Source: BMC Infect Dis. 2024 Mar 15;24:319. doi: 10.1186/s12879-024-09198-z (PMC10943806; doi:10.1186/s12879-024-09198-z)

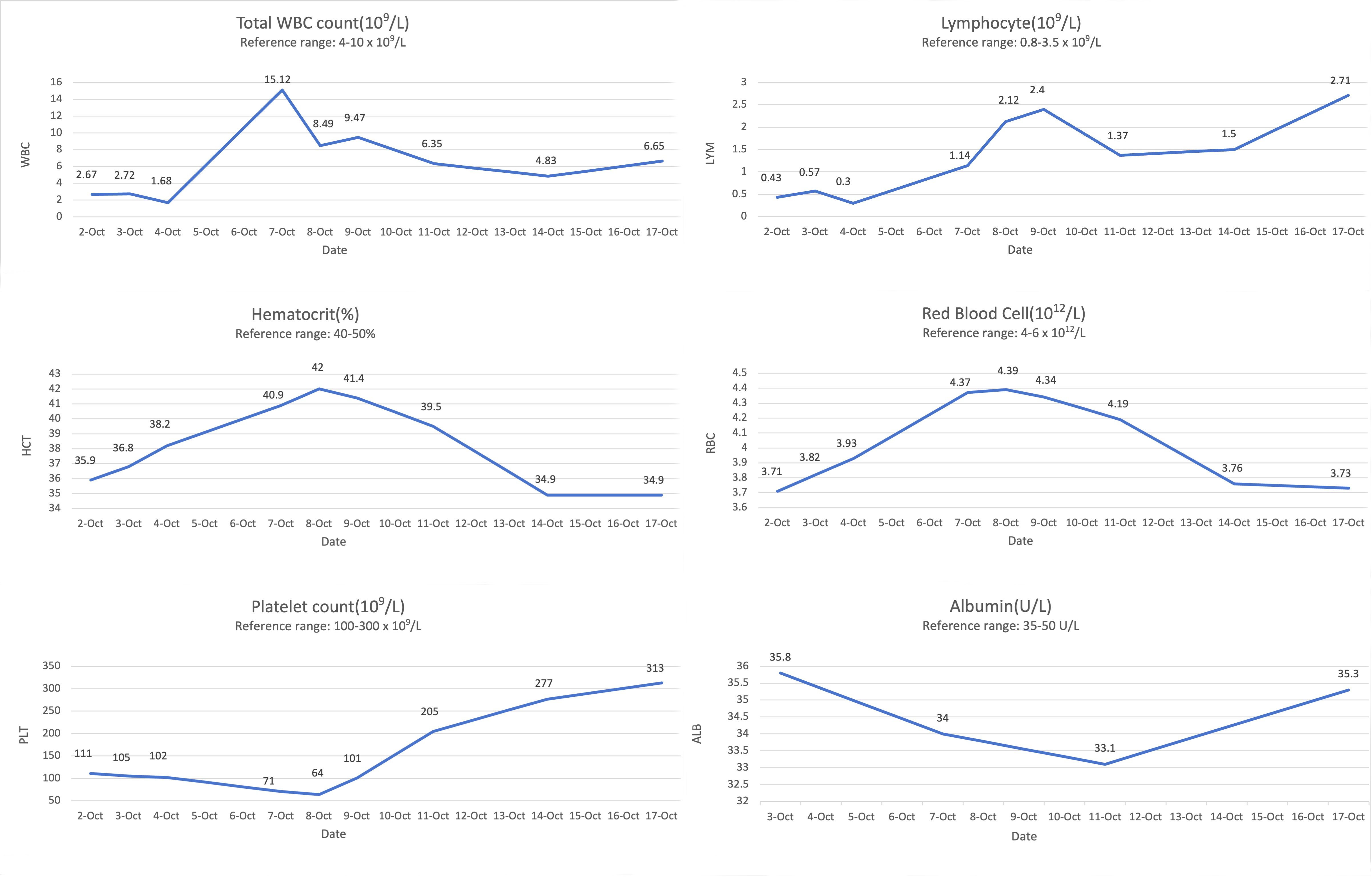

Supplement: Supplementary file 3 — Supplementary Material 3 [file 12879_2024_9198_MOESM3_ESM.png]
